# Alterations in metal homeostasis occur prior to canonical markers in Huntington disease

**DOI:** 10.1038/s41598-022-14169-y

**Published:** 2022-06-20

**Authors:** Anna C. Pfalzer, Yan Yan, Hakmook Kang, Melissa Totten, James Silverman, Aaron B. Bowman, Keith Erikson, Daniel O. Claassen

**Affiliations:** 1 Department of Neurology, Vanderbilt University Medical Center, 1611 21st Avenue South, Suite 1532, Nashville, TN 37232 USA; 2 Department of Biostatistics, Vanderbilt University Medical Center, Nashville, TN USA; 3 Department of Nutrition, University of North Carolina-Greensboro, Greensboro, NC USA; 4 School of Health Sciences, Purdue University, West Lafayette, IN USA

**Keywords:** Cellular neuroscience, Diseases of the nervous system, Basal ganglia

## Abstract

The importance of metal biology in neurodegenerative diseases such as Huntingtin Disease is well documented with evidence of direct interactions between metals such as copper, zinc, iron and manganese and mutant Huntingtin pathobiology. To date, it is unclear whether these interactions are observed in humans, how this impacts other metals, and how mutant Huntington alters homeostatic mechanisms governing levels of copper, zinc, iron and manganese in cerebrospinal fluid and blood in HD patients. Plasma and cerebrospinal fluid from control, pre-manifest, manifest and late manifest HD participants were collected as part of HD-Clarity. Levels of cerebrospinal fluid and plasma copper, zinc, iron and manganese were measured as well as levels of mutant Huntingtin and neurofilament in a sub-set of cerebrospinal fluid samples. We find that elevations in cerebrospinal fluid copper, manganese and zinc levels are altered early in disease prior to alterations in canonical biomarkers of HD although these changes are not present in plasma. We also evidence that CSF iron is elevated in manifest patients. The relationships between plasma and cerebrospinal fluid metal are altered based on disease stage. These findings demonstrate that there are alterations in metal biology selectively in the CSF which occur prior to changes in known canonical biomarkers of disease. Our work indicates that there are pathological changes related to alterations in metal biology in individuals without elevations in neurofilament and mutant Huntingtin.

## Introduction

Essential trace metals play a vital role in several metabolic processes throughout the body and brain. Although their concentrations are lower compared to more bulk elements like calcium and sodium, they are necessary for the proper function and structure of many proteins. In fact, approximately 10% of human genes contain zinc (Zn)-finger domains^1^. The most abundant trace elements in human body are Iron (Fe), Zn, Copper (Cu), and manganese (Mn). These compounds have been shown to regulate mitochondrial function^2^, oxidative stress^3^, inflammation, synaptic signaling, cell signaling, glycobiology^4,5^, neurotransmitter synthesis and protein aggregation^6^. Both intracellular and extracellular essential metals are tightly regulated because deficiencies and excesses in result in detrimental effects on biological systems. Regulation predominantly occurs at the level of the gut which prevents dietary over-exposures through absorption and instances where toxic accumulations of metals occur typically bypass gastrointestinal regulation. For instance, over-exposure to manganese through various occupations results in a Parkinsonian-like condition called Manganism^7^. Copper accumulation in the brain due to Wilson’s disease results in involuntary movements and cognitive impairment^8^. Conversely, deficiencies in copper seen in Menke’s disease is also associated with cognitive and motor impairments^9^.

The role of essential metals specifically in the context of Huntington Disease (HD) has been explored in in vivo and in vitro models of disease. There is a clear and consistent interaction between Mn and mutant Huntingtin (HTT) pathobiology in cell and rodent models. Manganese (Mn) exposure in HD cells can correct deficits in metabolic pathways implicated in HD pathology such as autophagy^10^ and insulin signaling^11^. Mn exposure can also correct abnormalities in the striatal urea cycle in HD rodents^12^. We also observe global suppression of transcriptomic and metabolomic response to Mn in the same rodent model^13,14^—suggesting impairments in Mn trafficking in HD. Iron also accumulates in post-mortem HD brains as well as in HD animal models^2^. More recently, iron accumulation was shown using MRI imaging in pre-manifest HD individuals^15^. Iron chelation improves molecular and behavioral indicators of disease^16^. Alterations in Cu homeostasis has received considerable attention. Aberrations in Cu homeostasis have been observed in cell and rodent models of HD^6^ as well as in cerebrospinal fluid^17^ and post-mortem tissue^18^. Further, Xiao et al. found a direct interaction between elemental Cu and mutant HTT aggregates^6^ and that increases in intracellular Cu lead to increased mHTT aggregation^6^. Clinical application of these findings were tested in a clinical trial designed to investigate the effect of a Cu chelator on clinical outcomes in motor manifest HD, though this study did not meet its primary endpoint^19^.

While there is consistent evidence for alterations in metal biology in HD, there are many unanswered questions regarding the stability of metal homeostasis and the timing over which these alterations take place. The establishment of HDClarity, a large biofluid collection initiative, has provided access to CSF and plasma samples from individuals with HD at different clinical stages of progression. Here we assessed levels of key metals (Cu, Fe, Mn and Zn) in CSF and plasma in HD Clarity samples. Our goal was to identify the timing and stability of changes in central and systemic metal biology in HD compared to canonical markers of disease.

## Results

### Clinical demographics

Pre-manifest participants were significantly younger than the control and manifest groups as expected. Late manifest participants were significantly older than the other three study groups as shown in Table 1, also as expected. There were no significant differences in trinucleotide C-A-G repeat length among the HD study groups (Table 1). Among the behavioral and functional assessments, total functional capacity (TFC) and symbol digit modality test (SDMT) were similar between the Control and PRE groups with significant reductions in TFC in MAN and LATE groups (Table 1). Performance on the Stroop Word Reading (SWR) and total motor score (TMS) assessment incrementally decreased with disease progression (Table 1). The composite Unified Huntington Disease Rating Scale (cUHDRS) is an indicator of disease stage using assessments of cognitive capacity (SDMT, SWR), motor function (TMS) and functional capacity for activities of daily living (TFC). cUHDRS score decreases with disease severity, where a negative value is indicative of a more progressed patient compared to an individual with a positive cUHDRS score. cUHDRS scores do not significantly differ between the Control and PRE group; whereas there is a progressive decline between PRE, MAN and LATE (Table 1).

**Table 1:** Participant baseline demographics by disease stage.

	Control (n = 12)	Pre-manifest (n = 16)	Manifest (n = 16)	Late manifest (n = 16)
Age	47.75 ± 10.81^a^	39.125 ± 9.78^b^	47.0625 ± 7.34^a^	57.6875 ± 8.74^c^
Sex (M,F)	7,5	8,8	7,9	9,7
CAG	19.75 ± 3.89^a^	42.4375 ± 1.46^b^	43.75 ± 1.77^b^	43.8125 ± 1.87^b^
TFC	12.92 ± 0.29^a^	12.625 ± 0.62^a^	10.25 ± 3.34^b^	5.0625 ± 4.25^c^
SDMT	55.67 ± 10.05^a^	52.75 ± 15.93^a^	34.25 ± 13.89^b^	21.33 ± 16.09^c^
SWR	107.75 ± 17.51^a^	84.1875 ± 28.03^b^	62.4375 ± 19.17^c^	43.82 ± 29.10^d^
TMS	1.42 ± 3.70^a^	6.3125 ± 8.93^b^	29.0625 ± 22.39^c^	58.01 ± 29.97^d^
cUHDRS	17.71 ± 1.72^a^	15.80 ± 2.73^a^	10.30 ± 4.99^a^	5.80 ± 6.76^b^

Differences in baseline demographic variables were identified using a 1-way ANOVA with post-hoc testing after determination of a significant main effect (p < 0.05). Participant age is reported in years as well as the proportion of males to females (M,F). The number of trinucleotide repeats (CAG) as well as performance on total functional capacity (TFC), symbol digit modality test (SDMT), stroop word reading (SWR), total motor score (TMS) and the compositive United Huntington Disease Rating Scale (cUHDRS) scores are reported here as mean ± S.D and values that do not significantly differ share a superscript.

### Metal stability

The stability of metal homeostasis was assessed in Control and PRE groups by comparing baseline to the 4–8 week follow up CSF and plasma metal levels (Table 2). Interestingly, there were no differences between baseline and follow up CSF or plasma metal levels in Control participants but in the PRE group, CSF Cu and plasma Zn (highlighted in grey) were significantly altered at the follow up visit compared to baseline.

**Table 2:** Baseline and Follow-up Cerebrospinal Fluid and Plasma Metals in Control and Pre-manifest Participants.

	Control (BL)	Control (FU)	Pre-manifest (BL)	Pre-manifest (FU)
CSF Mn (ug/L ± S.D)	7.20 ± 6.47^a^	4.44 ± 4.93^a^	7.23 ± 3.41^a^	13.21 ± 9.06^a^
CSF Cu (ug/L ± S.D)	14.32 ± 8.80^a^	16.47 ± 10.30^a^	27.45 ± 23.87^a^	42.52 ± 43.51^b^
CSF Fe (ug/L ± S.D)	111.07 ± 66.93^a^	114.45 ± 53.41^a^	192.71 ± 92.22^a^	162.12 ± 107.2^a^
CSF Zn (ug/L ± S.D)	207.41 ± 65.85^a^	270.11 ± 119.68^a^	171.29 ± 65.45^a^	191.89 ± 76.57^a^
Plasma Mn (ug/L ± S.D)	16.77 ± 4.23^a^	18.27 ± 4.80^a^	18.66 ± 8.94^a^	16.85 ± 7.97^a^
Plasma Cu (ug/dL ± S.D)	121.76 ± 19.38^a^	124.13 ± 41.63^a^	139.84 ± 41.47^a^	157.79 ± 44.08^a^
Plasma Fe (ug/dL ± S.D)	182.31 ± 74.04^a^	186.24 ± 59.89^a^	172.15 ± 64.16^a^	171.84 ± 58.10^a^
Plasma Zn (ug/L ± S.D)	118.26.3 ± 78.20^a^	178.52 ± 102.45^a^	178.30 ± 116.70^a^	68.54 ± 54.36^b^

Differences between baseline and follow up cerebrospinal fluid and plasma metal levels in control and pre-manifest participants. Differences were examined using Mann–Whitney tests between visits and not explored across genotypes. Baseline (BL) and 4–8 week follow up (FU) cerebrospinal (CSF) and plasma manganese (Mn), Copper (Cu), Iron (Fe), Zinc (Zn) were measured using GF-AAS and shown here as mean ± S.D and values that do not significantly differ share a superscript.

### Metals elevated in early HD

**Figure 1:**
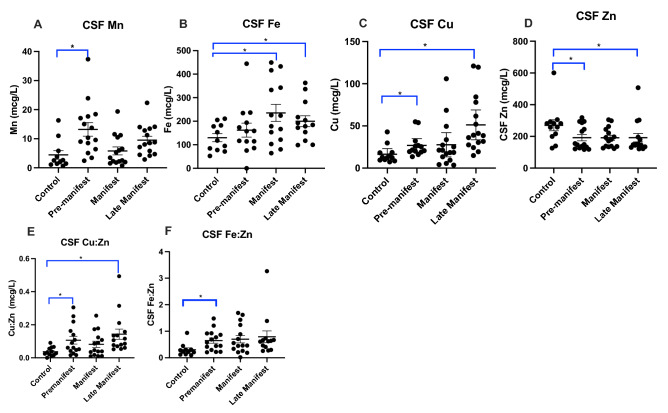
Cerebrospinal fluid (CSF) (**A**) manganese (Mn), (**B**) iron (Fe), (**C**) copper (Cu), (**D**) zinc (Zn) metal levels with the ratio of (**D**) Cu:Zn and (**E**) Fe:Zn between control and HD participants analyzed using 1-way ANOVA with post-hoc testing after determination of significant (p < 0.05) main effects. Data are shown as individual data points with the mean ± sem. An asterisk indicates a p-value < 0.05.

CSF Cu, Mn and Fe significantly increase in HD (Fig. 1A–C) compared to CSF Zn which decreases in HD samples. CSF Cu is significantly elevated in PRE and LATE participants compared to Control (Fig. 1C); in contrast, CSF Zn is significantly reduced in PRE and LATE participants compared to Control (Fig. 1D). CSF Fe is increased in MAN and LATE stage groups compared to Control. It is noteworthy that there were no significant correlations between CSF metal levels and age (data not included). It is well-known that Cu and Zn, Fe and Zn, and Mn and Fe are regulated in opposite directions by oxidative stress^20^ and as a result, the ratio of Cu:Zn, Fe:Zn and Mn:Fe could be a more robust indicator of early-onset pathology^20^ in HD. The ratio of CSF Cu:Zn and CSF Fe:Zn are elevated in the PRE and LATE study arms compared to Control (Fig. 1E,F). We found no significant correlations between CSF metal levels and CAG repeat length or CAP-score (Supplemental Table 1) and no relationship between metals and cUHDRS after adjusting for CAG repeat length (data not shown).

**Figure 2:**
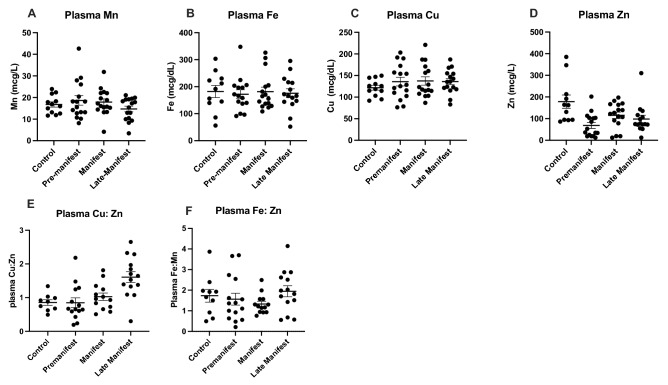
Plasma (**A**) manganese (Mn), (**B**) iron (Fe), (**C**) copper (Cu), (**D**) zinc (Zn) metal levels with the ratio of plasma (**E**) Cu:Zn and (**F**) Fe:Zn between control and HD participants analyzed using 1-way ANOVA with post-hoc testing after determination of significant (p < 0.05) main effects. Data are shown as individual data points with the mean ± sem.

Interestingly, the plasma showed no significant differences between Control and HD metal levels or the levels of metal ratios between Cu, Fe and Zn (Fig. 2A–G).

**Figure 3:**
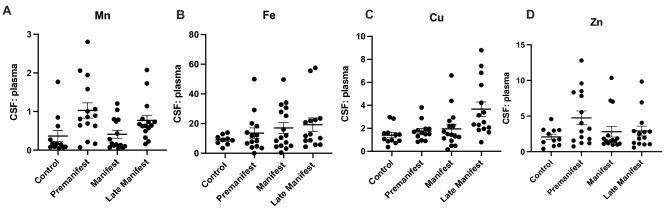
The ratio of cerebrospinal fluid (CSF) and plasma (**A**) manganese (Mn), (**B**) Iron (Fe), (**C**) Copper (Cu), and (**D**) Zinc (Zn) in control and HD participants. Differences in CSF: plasma metal ratio were analyzed using a 1-way ANOVA with post-hoc testing after determination of significant (p < 0.05) main effects. Data are shown as individual data points with the mean ± sem.

The ratio of CSF to plasma metal levels has previously been implicated as a marker of blood–brain barrier integrity. The ratio of CSF:plasma Cu significantly increases with disease stage (Fig. 3C) although individual pair-wise comparisons did not reach statistical significance.

**Figure 4:**
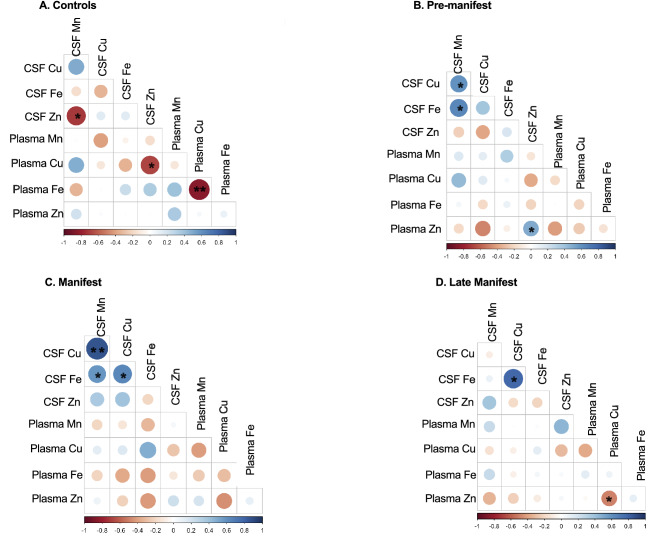
Correlation matrix between CSF and plasma metals in (**A**) Controls, (**B**) Pre-manifest, (**C**) Manifest and (**D**) Late Manifest HD participants. The size and color of the circle indicate the strength and direction of the correlation. Shades of red indicate negative correlations whereas blues indicate positive correlations. An asterisk (*) indicates p < 0.05 and (**) indicates p < 0.01.

We observed no significant correlations between CSF and plasma metals across all samples (data not shown). We next investigated the potential for this relationship to be disease specific and found that disease (stage) impacts the relationship between CSF and plasma metals (Fig. 4). Specifically, CSF Mn x CSF Zn, CSF Zn x plasma Cu, and plasma Fe x plasma Cu are all significantly negatively correlated in Control participants whereas none of these correlations exist in PRE, MAN or LATE study arms (Fig. 4). CSF Mn x CSF Cu, and CSF Mn x CSF Fe are both positively correlated in PRE and MAN (Fig. 4B,C) whereas this relationship does not exist for Control or LATE stage participants (Fig. 4A,D). A significant negative correlation appears in LATE stage participants between plasma Zn x plasma Cu (Fig. 4D).

### Metal levels change prior to elevations in biomarkers

**Figure 5:**
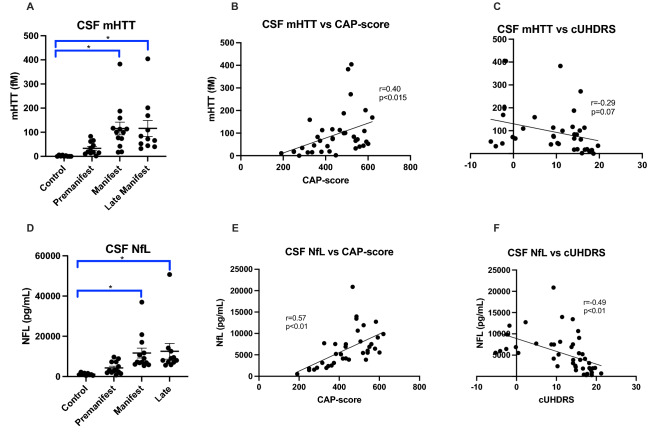
Cerebrospinal fluid mutant Huntingtin (mHTT) and Neurofilament light (NfL) in HD participants. CSF mHTT significantly increase with HD stage (**A**) and positively correlates with CAP-score (**B**) and negatively correlates with the composite Unified Huntington Disease Rating Scale (cUHDRS) (**C**). CSF NfL significantly increases with HD stage (**D**), and positively correlates with CAP-score (**E**) and negatively correlates with cUHDRS (**F**). An asterisk (*) indicates a p < 0.05 for post-hoc comparisons. Data are shown as individual data points with the mean ± sem .

**Figure 6:**
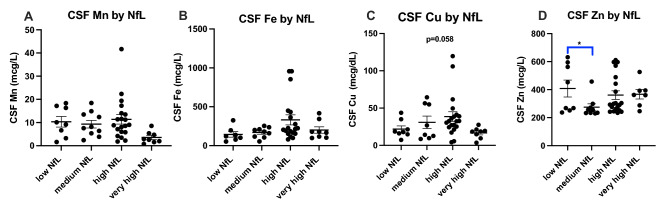
Cerebrospinal fluid (**A**) Mn, (**B**) Fe, (**C**) Cu and (**D**) Zn by Neurofilament light (NfL) in HD participants. NfL was categorized into low (0–1500 pg/mL), medium (1501–5000 pg/mL), high (5001–10,000 pg/mL) and very high (10,000 + pg/mL). Differences in CSF metal level by NfL accumulation was assessed using 1-way ANOVA with post-hoc testing after determination of a significant (p < 0.05) main effect. An asterisk (*) indicates significant (p < 0.05) two-way comparison. Data are shown as individual data points with the mean ± sem.

**Figure 7:**
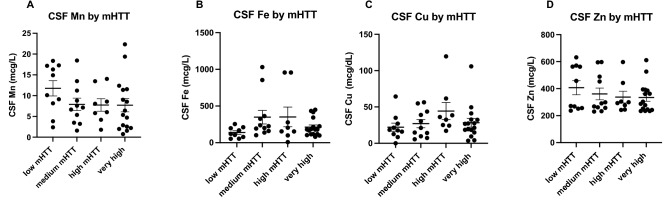
Cerebrospinal fluid metal levels by mutant Huntingtin (mHTT) accumulation in HD participants. mHTT was categorized into low (0-10fM), medium (11–49 fM), high (50–100 fM), very high (101 + fM). Differences in CSF (**A**) Mn, (**B**) Fe, (**C**) Cu and (**D**) Zn by mHTT concentration were assessed using 1-way ANOVA. Data are shown as individual data points with the mean ± sem.

As expected, mutant Huntingtin (mHTT) levels increase with disease stage (Fig. 5A), disease burden (Fig. 5B) and clinical severity (Fig. 5C). Similarly, Neurofilament light (NfL) levels also increase with disease stage (Fig. 5D), disease burden (Fig. 5E) and clinical severity (Fig. 5F). Interestingly, mHTT and NfL levels were not significantly different between our Control and PRE groups (Figs. 5A,D). We examined how increases in mHTT and NfL related to CSF metal levels (Figs. 6 and 7). CSF Cu levels increase with modest elevations in NfL, but are reduced in individuals with the highest levels of NfL (ANOVA p-value: 0.058; Fig. 6C). A reverse trend appears in CSF Zn, with reductions in Zn corresponding to modest elevations in NfL (Fig. 6D). We found no clear indication that CSF metal levels were impacted by mHTT levels (Fig. 7).

## Discussion

The role of essential metals in neuronal health has been well-established^21^ with deficiencies and excesses both resulting in neurological symptoms which include cognitive deficits and involuntary movements^22^. Metal levels are elevated in post-mortem tissue in HD^23^. Several in vivo and in vitro studies in HD disease models demonstrate that alterations in metal biology impact molecular pathways implicated in HD pathology^10,12,13^. There is also a direct interaction between elemental Cu and exon 1 of mutant Huntingtin (HTT) protein^6^. Despite these observations directly linking metal biology to HD pathology, the timing and stability of changes to the metallome are unknown as well as whether changes in the CNS are recapitulated in blood. Our work outlined below begins to address these unanswered questions.

Here, we assessed CSF and plasma metal levels in a cohort of control and HD participants, and examined relationships between metals and HD biomarkers in a sub-set. Pre-manifest participants were significantly younger in age compared to control, but had similar scores on clinical indicators of cognitive function and quality of life. As expected, cognitive and motor function decline with disease progression. Prior to this decline, we note elevations in CSF Mn and Cu, and reductions in CSF Zn. That is, we infer the timing of these changes based on our findings in pre-manifest participants, but not manifest or late cohorts. Furthermore, the pre-manifest metallome (particularly CSF Cu and plasma Zn) has substantial differences between baseline and follow-up visits—indicating potential differences in metal homeostasis and stability in early HD. We are unable to differentiate whether this lack of metal homeostasis may reflect pathology caused by early mHTT or some other biological or environmental variable specific to HD. Additionally, we saw no elevation in CSF Fe in premanifest HD individuals. While a recent study that found increases in striatal iron using MRI imaging^15^, our results suggest that intracellular or parenchymal increases in metals are not always reflected in extracellular assays (e.g. CSF). There was no correlation between age, and metal levels (CSF or plasma). These elevations in CSF Cu (24-47 μg/L) are not as substantial as that seen in Wilson’s disease, ~ 76 μg/L^22^. We do not observe these same alterations in Mn, Cu and Zn in plasma although we do find that the CSF:plasma ratio for Mn and Cu are significantly elevated in HD compared to controls (Fig. 3). Taken together, these findings demonstrate that there are early alterations in metal homeostasis in the CSF, not observed in the plasma. It is possible that plasma metal homeostasis is more tightly regulated at the level of the gut to prevent drastic elevations in metal levels. Nevertheless, these CSF-specific changes may reflect changes to the integrity of the blood–brain barrier (BBB) or metal transport across the blood–brain barrier, which is noted to be impaired early in disease in rodent models^24^.

Additionally, we find that the interactions among essential metals changes with disease progression. Because of essential metal interdependency, dys-homeostasis of a single metal will result in aberration dys-homeostasis of others. For instance, increased Cu can replace Zn on Zinc-dependent enzymes which alters the functional status of those proteins^25^. Our results show a negative correlation between CSF Mn and Zn only in controls. Previous research demonstrates that increases in Mn levels are associated with reductions in Zn under control conditions. This suggests that CNS Mn- or Zn-dependent transport is also altered early in HD.

Perhaps the most striking finding relates to our observed changes in CSF metals occur prior to elevations in canonical markers of HD. Specifically, the early changes in CSF Cu, Mn and Zn levels pre-date changes in mHTT and NfL in the pre-manifest participants compared to control. We interpret these findings with the consideration that only a subset of our metal samples had biomarker data available at the time of this manuscript. However, these findings suggest that subtle increases in mutant Huntingtin result in robust alterations in essential metal regulation. It should also be noted that these elevations in CSF metals appear fairly selective to premanifest disease and thus, may not be an ideal biomarker for later stage studies of HD. This might also explain why there were no significant relationships between metal level and markers of disease progression. There are several known physiologic mechanisms by which excess Cu exerts detrimental effects—which may contribute, in part, to the pathology of HD. The toxicity of Cu depends largely upon whether Cu is bound to transport proteins or a free ion. Unfortunately, our methodology does not allow us to differentiate between free and bound metals and thus, we cannot interrogate the possibility that elevations in metal levels reflect increases in free reactive ion species. It is known that excesses of extracellular free Cu ions dramatically increase oxidative stress through its role in free radical regulation^26^. Free Cu initiates the production of free radicals through the Fenton reaction which produces reactive hydroxyl groups^26^. Copper exposure also stimulates the secretion of pro-inflammatory cytokines such as IL-1, IL-4, TNFα in the brain and blood^27^. These same cytokines are elevated in blood of Huntington Disease patients^28^. Lastly, there is evidence that Cu (as well as Zn) regulates neurotransmission in the brain. Studies examining the role of copper in neurotransmitter secretion found that substances which induce Cu release from cells also induce the synthesis and secretion of the primary inhibitory neurotransmitter GABA^29^. Copper release has also been linked to NMDA receptor activation—where localization of the copper transporter to the plasma membrane activates NMDA receptors^30^. Imbalances to GABA homeostasis have been clearly delineated in Huntington Disease with the striatum being one of the most densely connected areas to GABA-ergic neurons^31^. It is feasible that elevations in Cu and reductions in Zn participate in the pathogenesis of HD through their involvement in the management of reactive oxygen species, inflammation and neurotransmission.

Although we demonstrate clear elevations in CSF metals prior to elevations in canonical markers of disease, the cause of these *extracellular* changes and their *intracellular* consequences remain unclear. More so, it is unknown whether the extracellular space recapitulates what is occurring intracellularly. Based upon the known interaction between Cu and mutant Huntingtin aggregates, we postulate that the accumulation of aggregates with bound Cu accelerates neuronal death in two ways: (i) Cu increases mutant Huntingtin aggregation and (ii) deficiencies in Cu-dependent biological processes due to sequestration by mutant Huntingtin. Interestingly, there is considerable overlap between the molecular mechanisms implicated in HD pathology and Cu-dependent biological processes: mitochondrial function via cytochrome C oxidase, dopamine excess via dopamine β-hydroxylase, and oxidative stress via superoxide dismutase 1. Our observed elevations in CSF Cu early in disease may not recapitulate intracellular levels, in fact, we propose that in HD, Cu is sequestered by mutant HTT and thus creates conditions of intracellular Cu deficiency despite elevations in the extracellular space.

It is also necessary to acknowledge the limited sample size for this study, in particular, the lack of availability of biomarker data for a sub-set of samples. The limited sample size and the lack of detailed demographic information in participants should be taken into consideration when interpreting the clinical significance of these findings.

In sum, our work provides important insights into metal biology under normal homeostatic mechanisms as well as alterations in these mechanisms in the context of HD. We report here that CSF Cu, Mn and Zn are altered prior to established disease biomarkers. Our findings provide a strong scientific premise to further explore: (i) the potential for CSF metals to be early biomarkers of HD and (ii) the mechanistic link between Cu and Zn and mHTT and how alterations in these intracellular/extracellular metal levels contribute to neuronal pathology. A closer examination of the role between CSF metals and mHTT would determine whether Cu could be a novel biomarker in early HD pathology or Cu regulation should be explored again in the context of premanifest HD as a potential therapeutic target. Together, these investigations will likely inform the new phase of clinical investigations.

## Methods

### Samples

Plasma and cerebrospinal fluid (CSF) were collected from 60 participants as part of the CHDI HDClarity study. Samples were collected and processed according to a standard protocol^32^. Briefly, samples were collected between 8 and 10am after participants fasted overnight. After processing, samples were immediately stored at -80C until distribution to investigators^33^. There were 16 pre-motor manifest (PRE), 16 manifest (MAN) and 16 moderate-late (LATE) manifest HD and 12 control participants. Disease stage was determined using the diagnostic confidence level (DCL) which categorizes individuals’ based solely on motor symptoms^34^, length of CAG expansion and burden of pathology^35^ calculated from (CAG expansion—35.5) x Age. Control participants were individuals without a known history of Huntington Disease (HD). All HD participants have a CAG expansion of ≥ 40. PRE individuals were not motor manifest as indicated by a DCL of < 4 and a burden of pathology of > 250. MAN participants had a DCL = 4 and a total functional capacity (TFC) between 7–13. The LATE group had all the above criteria for MAN and a TFC score between 0–6. Repeat CSF and blood samples collected 4–8 weeks after a baseline visit are provided for all control and PRE participants. Participant age and gender are reported here. Three participants (2 PRE, 1 MAN) were taking supplemental vitamins however, their metal levels were similar to those in their corresponding participant group and thus, the data from these participants are included. Participants were not actively taking any investigational drug for HD (and had not for at least 30 days prior to screening). Basic demographics like age and gender are reported here in addition to participants scores on a battery of cognitive, behavioral and motor assessments including the symbol digit modality test (SDMT), Stroop Word Reading (SWR), total functional capacity (TFC) and total motor score (TMS). Schobel et al. recently proposed a novel indicator of clinical severity, the composite Unified Huntington Disease Rating Scale (cUHDRS) which incorporates a participants performance on the four aforementioned functional, cognitive and motor tasks: TMS, SWR, SDMT and TFC^36^.

### Metals

Plasma and CSF iron (Fe), manganese (Mn), copper (Cu) and zinc (Zn) concentrations were measured with graphite furnace atomic absorption spectrometry (GFAAS, Varian AA240, Varian, Inc., Palo Alto, CA). Fifty microliters of plasma and CSF were digested in ultrapure nitric acid (1:10 wt/vol dilution) for 48–72 h in a sand bath (60 °C); 50 μL of digested sample was brought to 1 mL of total volume with 2% nitric acid and analyzed for Mn, Fe, Cu and Zn. CSF samples were diluted 1:4 for Cu and Mn and diluted 1:32 or 1:64 for Fe and Zn. Plasma samples were diluted 1:20 for Mn, 1:40 for Cu, 1:100 for Fe and up to 1:1000 for Zn. The dilutions for these samples were based upon a standard curve specific to each metal. Metal levels were evaluated in triplicate for each sample with the mean value reported here for analyses. A bovine liver (NBS Standard Reference Material, USDC, Washington, DC) was digested in ultra-pure nitric acid and used as an internal standard for analysis.

### Biomarkers

An interim analysis of known HD biomarkers was conducted by a research team at CHDI which analyzed Neurofilament light (NfL), mutant Huntingtin (mHTT), total Huntingtin (totHtt), total protein (totPro) and Hemoglobin (Hb). Specific information regarding assays are available on the CHDI website^37^. As this was an interim analysis, cerebrospinal fluid biomarker data was only available for a sub-set of samples (n = 9 control, n = 12 pre-manifest, n = 13 manifest and n = 11 late manifest). There were no samples which had both a baseline and follow-up visit and to date, no biomarker data in blood. Data are reported here as picograms/milliliter (pg/mL) of NfL or femtomolar (fM) mHTT adjusted by total protein levels (i.e. NfL/total protein and mHTT/total protein) to account for differences in CSF protein levels. Associations between essential metals and biomarkers were analyzed by grouping mHTT and NfL into low, medium, high and very high categories based upon previous categorizations.

### Statistics

We utilized a parametric, univariate ANOVA to analyze differences in (baseline participant demographics and assessments: age, CAG repeat length and scores for total functional capacity, symbol digit modality test, stroop word reading, total motor score from the Unified Huntington Disease Rating Scale (UHDRS) and composite UHDRS (cUHDRS) (Table 1). We utilized non-parametric models for all metal and biomarker analyses to prevent assumptions of normality as previous work examining metals in biofluids display normal^38^ and non-normal distributions^39^. One sample from the MAN and LATE group was dropped for all metal analyses. A second sample was dropped from the CSF Fe analyses has the levels of Fe were ‘undetectable’. A univariate Kruskal–Wallis ANOVA was used to identify differences in CSF and plasma metal (ratio) levels between disease groups as well as differences between levels of the HD biomarkers mutant HTT and NfL (more detailed results provided in Supplemental Table 1). Sex was not a significant co-variate in the relationship between CSF and plasma levels and HD stage. CSF and plasma data are reported as the mean standard error of the mean (sem). Tukey’s post-hoc testing was completed after determination of a significant (p < 0.05) main effect. Group values which *do not* significantly differ share the same superscript. To investigate the stability of metal homeostasis, we only examined the effect of time within a genotype (i.e. baseline vs follow up) using Mann–Whitney tests. Correlations between CSF and plasma metal levels were assessed using Spearman correlations for non-parametric data with Spearman correlation coefficients and p-values adjusted for multiple comparisons reported. Correlations between metal levels and cUHDRS were examined after adjusting for CAP and CAG-repeat length.

### Ethics

There samples were provided by CHDI as de-identified samples with select pieces of medical and clinical information which did not pose additional risk for participant identification. The molecular work outlined in this manuscript was approved by the Vanderbilt University Medical Center Internal Review Board (IRB# 191615) and adhered to all relevant guidelines and regulations. Informed Consent was obtained at each HDClarity study site prior to conducting any study procedures performed in accordance with the Declaration of Helsinki.

## Supplementary Information

Supplementary Information 1.
