# Supplementary figures and images for: Alterations in metal homeostasis occur prior to canonical markers in Huntington disease
Source: Sci Rep. 2022 Jun 20;12:10373. doi: 10.1038/s41598-022-14169-y (PMC9209499; doi:10.1038/s41598-022-14169-y)

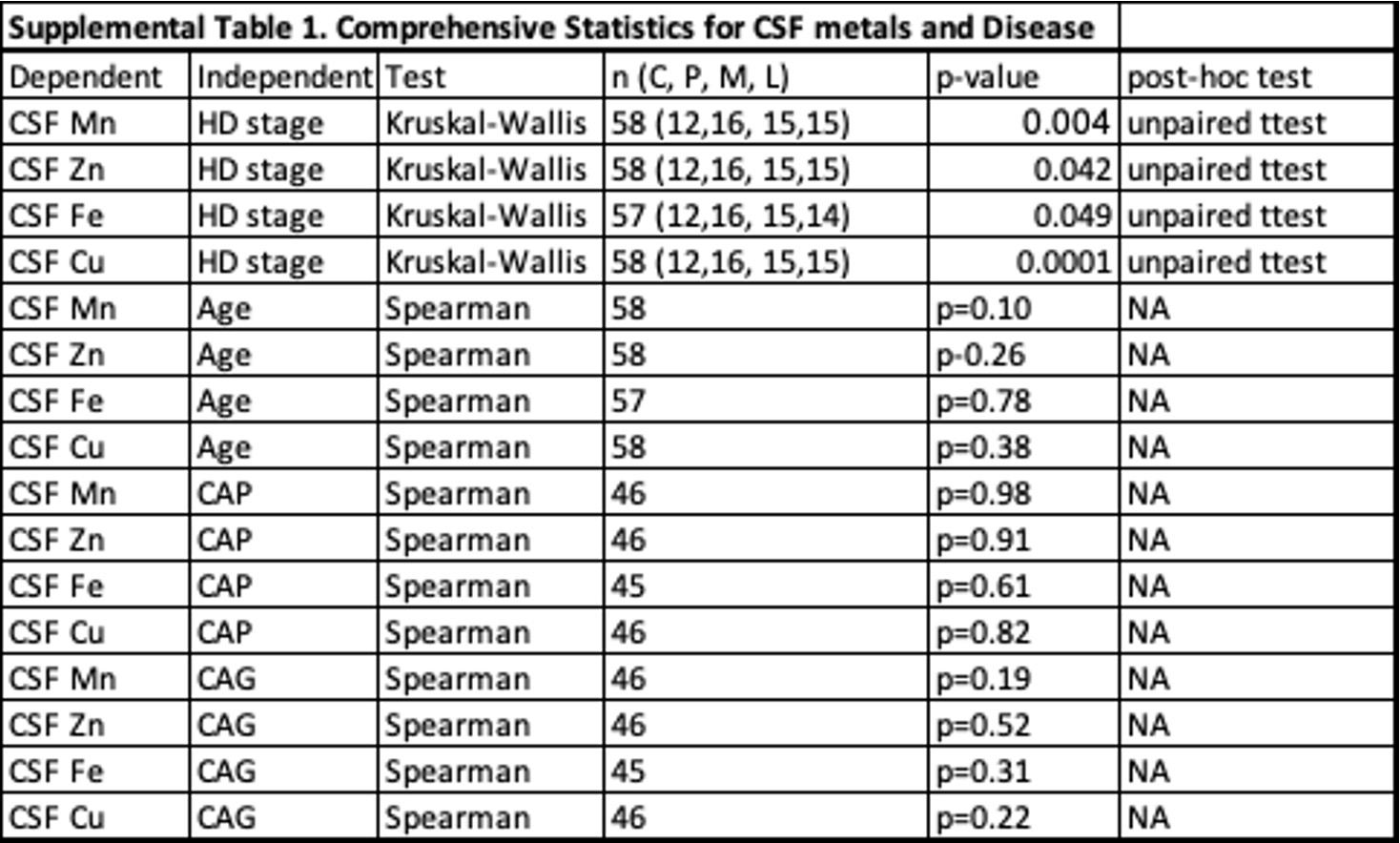

Supplement: Supplementary file 1 — Supplementary Information 1. [file 41598_2022_14169_MOESM1_ESM.jpg]
